# What are the effects of having an illness or injury whilst deployed on post deployment mental health? A population based record linkage study of UK Army personnel who have served in Iraq or Afghanistan

**DOI:** 10.1186/1471-244X-12-178

**Published:** 2012-10-24

**Authors:** Harriet J Forbes, Norman Jones, Charlotte Woodhead, Neil Greenberg, Kate Harrison, Sandra White, Simon Wessely, Nicola T Fear

**Affiliations:** 1 London School of Hygiene and Tropical Medicine, Keppel Street, London, WC1E 7HT, UK; 2 King’s Centre for Military Health Research, King’s College London, Weston Education Centre, 10 Cutcombe Road, London, SE5 9RJ, UK; 3 Academic Centre for Defence Mental Health, King’s College London, Weston Education Centre, 10 Cutcombe Road, London, SE5 9RJ, UK; 4 DASA Health Information, Defence Analytical Services and Advice (DASA), UK Ministry of Defence, Ensleigh, Bath, BA1 5AB, UK

**Keywords:** Mental Health, Military, PTSD, Alcohol use, Depression, Deployment

## Abstract

**Background:**

The negative impact of sustaining an injury on a military deployment on subsequent mental health is well-documented, however, the relationship between having an illness on a military operation and subsequent mental health is unknown.

**Methods:**

Population based study, linking routinely collected data of attendances at emergency departments in military hospitals in Iraq and Afghanistan [Operational Emergency Department Attendance Register (OpEDAR)], with data on 3896 UK Army personnel who participated in a military health study between 2007 and 2009 and deployed to Iraq or Afghanistan between 2003 to 2009.

**Results:**

In total, 13.8% (531/3896) of participants had an event recorded on OpEDAR during deployment; 2.3% (89/3884) were medically evacuated. As expected, those medically evacuated for an injury were at increased risk of post deployment probable PTSD (odds ratio 4.27, 95% confidence interval 1.80-10.12). Less expected was that being medically evacuated for an illness was also associated with a similarly increased risk of probable PTSD (4.39, 1.60-12.07) and common mental disorders (2.79, 1.41-5.51). There was no association between having an OpEDAR event and alcohol misuse. Having an injury caused by hostile action was associated with increased risk of probable PTSD compared to those with a non-hostile injury (3.88, 1.15 to 13.06).

**Conclusions:**

Personnel sustaining illnesses on deployment are just as, if not more, at risk of having subsequent mental health problems as personnel who have sustained an injury. Monitoring of mental health problems should consider those with illnesses as well as physical injuries.

## Background

Routinely collected data suggests around 20% of UK troops attended hospital whilst deployed (on a military operation) in Iraq between 2004 and 2006 [1]. Furthermore, between 2003 and 2009, over 6,900 UK military and civilian personnel were medically evacuated back to the UK from Iraq or Afghanistan [2]. On deployment, personnel can be in combat roles where they are in contact with the opposition or non-combat roles, where contact with the enemy is limited. Historically, a large-proportion of medical casualties and air-evacuations during military operations were related to illness and non-combat injury, particularly diarrhoeal disease [3], rather than combat injury, and the conflicts in Iraq and Afghanistan are no exception [2,4,5].

The mental health consequences of sustaining a physical injury during deployment, particularly injuries resulting from combat action, have been well-researched. Injury is well recognised as a risk factor for mental health problems, especially post-traumatic stress disorder (PTSD) in military [6] and non-military communities [7,8]. Injured personnel are often in the public eye, being focussed on by the media and charities. The impact of having an illness whilst deployed upon mental health however has not been well explored and analysis of illness has been largely confined to assessing its prevalence and operational impact [4]. This is surprising given that illness makes up a large proportion of casualties during operational deployment. The way in which illness can impact negatively on mental health is an area of growing interest in civilian populations [9] and may also be relevant to the military.

This study aims to tests the hypothesis that having an injury whilst deployed increases the risk of post deployment mental health problems whereas having an illness does not.

## Methods

### Study design

The study compared personnel presenting with an injury or illness at deployed military hospitals in Iraq or Afghanistan, with personnel not presenting with an injury or illness. The study linked cohort data from phase 1 and 2 of the King’s Centre for Military Health Research (KCMHR) Military Health Study with routinely collected data from the Operational Emergency Department Attendance Register (OpEDAR). Analysis was restricted to participants’ most recent deployment to Iraq or Afghanistan (referred to as ‘deployment’ from herein) because the KCMHR study collected data on most recent deployment and deployment specific factors could then be controlled for.

### Cohort study

Phase 1 and 2 of the KCMHR Military Health Study were the first and second phases of an ongoing cohort study of UK military personnel assessing physical and mental health consequences of deployment [10,11]. Phase 1 data were collected between June 2004 and March 2006 and phase 2 between November 2007 and September 2009. There were 10,272 participants at phase 1 (response rate was 59%) and 9984 participants at phase 2 (response rate was 56%), some of whom had been followed up from phase 1 and some newly recruited to ensure the sample remained representative of the UK military. Response was associated with older age, being female, being an officer and being a regular (the military categorises personnel into two engagement types: regulars, who are in full-time military employment, and reservists). There was no evidence that response was associated with mental health status.

#### Socio-demographic and deployment experiences

Socio-demographic characteristics and deployment experiences were taken from phase 2, or from phase 1 if the participant had not deployed between phase 1 and 2. Seven questions on traumatic deployment experiences which were common to both phase 1 and phase 2 questionnaires were as follows: did you ‘give aid to wounded’, ‘see personnel seriously wounded or killed’, ‘come under small arm/RPG fire’, ‘come under mortar/artillery fire/rocket attack’, ‘experience a landmine strike’, ‘experience hostility from Iraqi/Afghani civilians’ and ‘handle bodies’. Total number of traumatic deployment experiences was calculated and participants categorised as having none, 1–3 or ≥4 traumatic deployment experiences.

#### Health measures

Four measures of current health status collected at phase 2 of the KCMHR Military Health Study were included in the analysis. Probable PTSD was measured using the 17-item civilian version of the PTSD checklist (PCL-C) [12] using a cut-off score of 50 or more to define probable cases of PTSD. Symptoms of common mental disorders were measured with the 12-item General Health Questionnaire (GHQ-12) [13] using a cut-off of 4 or more to define cases of common mental disorder. Alcohol use was measured using the 10-item WHO Alcohol Use Disorders Identification Test (AUDIT) [14], using a cut-off of 16 to define alcohol misuse [15]. General health perception was rated using an item from the 36-item Short Form Health Survey as either poor, fair, good or excellent [16]; cases were those reporting ‘poor’ or ‘fair’ health.

### Operational emergency department attendance register (OpEDAR)

Illness and injury events occurring on deployment were supplied by the UK Ministry of Defence (MoD) via Defence Analytical Services and Advice (DASA). The data were gathered from three sources:

Operational Emergency Department Attendance Register (OpEDAR): A record of attendances to field hospitals on deployment [1]. Data were provided on date and location of attendance, diagnosis, cause (hostile or non-hostile), classification (e.g. psychiatric, musculoskeletal, respiratory) and disposal type (returned to unit, admitted to hospital, or medically evacuated to the UK). OpEDAR is completed by emergency department staff. Events on OpEDAR occurring in Iraq between February 2003 and April 2009, and in Afghanistan between August 2006 and December 2009 were available (OpEDAR data from Afghanistan reporting events occurring prior to August 2006 were not of sufficient quality to be included in the analysis).

NOTICAS Reports: Generated when a patient requires hospitalisation for a serious condition and relatives are notified [1]. Data were provided on date and location of attendance, diagnosis and primary cause (e.g. natural cause or enemy fire).

J97 health surveillance system: Routine data from any medical facilities in the UK or whilst overseas on deployment or training [17]. Data were provided on date and location of attendance, diagnosis, cause (hostile or non-hostile) and disposal.

The majority of events (505/531, 95.1%) were listed on OpEDAR, therefore, for the purposes of this paper, events will be referred to as an ‘event on OpEDAR’.

#### Classifying events on OpEDAR

Each event was classified as an injury or an illness (including both physical and psychiatric illnesses) using the diagnostic information provided, by one of the authors (HF) under guidance from NJ (an Army Nurse). There was insufficient information to classify events in more detail than ‘injury’ or ‘illness’, though injuries were classified as hostile and non-hostile.

For individuals with multiple events, the most severe event was selected for analysis, using the following hierarchy: injury resulting in a medical evacuation to the UK (most severe), illness resulting in a medical evacuation to the UK, injury resulting in hospital admission, illness resulting in hospital admission, injury resulting in being returned to unit, illness resulting in being returned to unit (least severe). Of 531 individuals with an event on OpEDAR, 108 had >1 event. The prevalence of mental health conditions did not differ significantly between those with single or multiple events [data not shown].

#### Study sample

**Figure 1:**
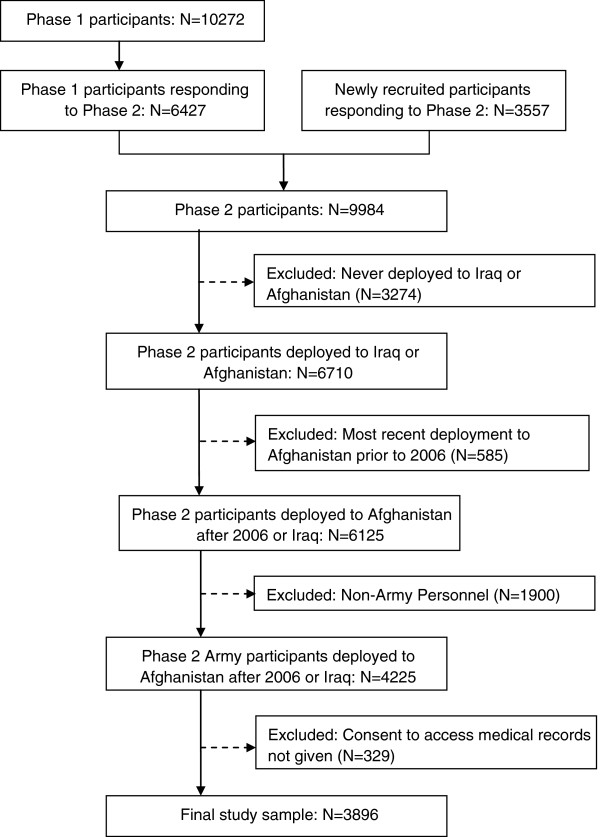
**Flow Diagram of participants in the study sample.**

Only those completing phase 2 of the KCMHR Military Health study were included. Of those 9984 individuals, the following were excluded; 3274 personnel never having deployed to Iraq or Afghanistan; 585 personnel whose most recent deployment was to Afghanistan before October 2006 (events on OpEDAR were incomplete for these operations); 1900 non-Army personnel (the majority of personnel deployed to Iraq or Afghanistan are Army and non-Army personnel have few injury or illness events [less than 2% of non-army personnel had an OpEDAR event]); and 329 personnel (7.8%) who did not consent to use of medical records. The final sample size was 3896 (Figure 1).

### Statistical analysis

The OpEDAR data were linked with the cohort data using a unique identifier. Logistic regression was used to calculate odds ratios (OR) and 95% confidence intervals (CIs) to identify socio-demographic or military factors associated with having an injury or illness event on OpEDAR. This helped assess their potential as confounders for the analyses of OpEDAR events and health outcomes. Socio-demographic or military characteristics showing association (P < 0.1) with an illness or injury event on OpEDAR in univariable analyses were included in the multivariable model.

Logistic regression was used to analyse the overall effect of having any OpEDAR event on health outcomes and subsequently OpEDAR events were assessed in greater detail looking at the effects of the type (i.e. illness or injury) and severity (using disposal status as a proxy) of the OpEDAR event on health outcomes. Additionally, the effect of a hostile, compared to a non-hostile injury, on mental health was tested. Confounders were identified if they substantially altered the OR [approximately 10%], adjusted for a-priori confounders.

All analyses adjusted for a-priori confounding factors, age, sex, rank and engagement status [11,18]. Analyses did not account for survey design as a sub-sample of the original cohort was used. Response weights were used in all analyses, to reduce non-response bias. Response weights were defined as the inverse probability of responding once sampled, driven by factors shown to predict response (sex, rank, engagement type, age) [10]. Analysis was undertaken using the statistical software package STATA (version 11.0).

#### Sensitivity analyses

As one event was selected for analysis via the hierarchy set out above, another analysis was run where illness took precedence over injury. As well as multiple events on OpEDAR, 260 personnel had OpEDAR events in Iraq or Afghanistan before their most recent deployment. An analysis was thus carried out adjusting for having had any event on OpEDAR before most recent deployment. As personnel presenting to in-field hospitals with psychiatric illnesses may be at increased risk of post-deployment mental health problems, an analysis was conducted excluding OpEDAR events classified as “psychiatric”. Finally, although previous history of mental health was not recorded at phase 2, some participants completed a phase 1 questionnaire, where mental health indicators were recorded. A sensitivity analysis was carried out adjusting for mental health status at phase 1, among personnel who completed both phase 1 and phase 2 and had either an OpEDAR event after phase 1 or no events. Personnel with phase 1 mental health problems were all those defined as a PTSD or common mental disorder case.

### Ethics approval

The study received ethics approval from the MoD’s research ethics committee (MODREC) and King’s College Hospital’s local research ethics committee. Participants gave informed consent before taking part.

## Results

The study sample comprised of 3896 Army personnel (see Figure 1) of which 13.8% (531/3896) had an event recorded on OpEDAR whilst on deployment and 2.3% (89/3884) were medically evacuated back to the UK (Table 1). The most common illness was gastrointestinal illness (45.7%) and the most common injuries were orthopaedic soft tissue injuries and musculo-skeletal injuries (40.8%). Of all injuries, 15.1% (33/261) were ‘hostile’.

**Table 1:** **Prevalence of illness and injuries occurring on deployment, by disposal type, among 3896 Army personnel**

**OpEDAR event**		**Participants with OpEDAR event**	**Participants with disposal type among those with OpEDAR event**
		**n/N (%)**	**n/N (%)**
**All events**	**Any hospital attendance**	531*/3896 (13.8)	-
	Returned to unit	232/3884 (6.0)	232/519 (44.8)
	Admitted	198/3884 (5.1)	198/519 (38.1)
	Medically evacuated	89/3884 (2.3)	89/519 (17.1)
**Illness events**	**All illness**	270**/3896 (6.8)	-
	Returned to unit	75/3890 (1.9)	75/264 (27.1)
	Admitted	151/3890 (3.9)	151/264 (58.9)
	Medically evacuated	38/3890 (1.0)	38/264 (14.1)
**Injury events**	**All injury**†	261***/3896 (6.9)	-
	Returned to unit	157/3890 (4.0)	157/255 (62.2)
	Admitted	47/3890 (1.2)	47/255 (17.8)
	Medically evacuated	51/3890 (1.3)	51/255 (20.0)

*12 OpEDAR events are missing ‘disposal’ information.

**6 illness events on OpEDAR are missing ‘disposal’ information.

***6 injury events on OpEDAR are missing ‘disposal’ information.

† 33 of 261 injuries (15.1%) were hostile (19 of these were medically evacuated, 11 admitted and 3 returned to unit).

Median time since deployment was 2.0 years (IQR 0.8-4.5 years). Those consenting to use of their medical records were older and were more likely to have left the military, compared to non-consenters [data not shown; see Additional file 1].

### Association between having an OpEDAR event and socio-demographic and military characteristics

Being aged 40 years or over was associated with having an illness event on OpEDAR (Table 2). Females and reservists had around twice the odds of having an illness and an injury event on OpEDAR. Officers were less likely to have an illness event recorded on OpEDAR. There was no association with role. There was, however, a graded response relationship between the number of ‘traumatic deployment experiences’ and the odds of illness and injury, with the odds increasing with the number of experiences.

**Table 2:** **Associations between having an illness or injury event on OpEDAR and socio-demographic and military characteristics among 3896 Army personnel**

	**Number ill on last deployment (%)**	**Unadjusted OR (95%CI)**	Adjusted OR* (95%CI)	**Number injured on last deployment (%)**	**Unadjusted OR (95%CI)**	Adjusted OR* (95%CI)
**Age group (years)**						
<25	45/657 (6.9)	1	1**	52/657 (7.9)	1	1***
25-29	56/832 (6.7)	0.96 (0.63-1.46)	1.05 (0.69-1.61)	56/832 (7.0)	0.88 (0.59-1.32)	0.91 (0.60-1.38)
		P=0.852	P=0.815		P=0.549	P=0.659
30-34	40/726 (5.4)	0.76 (0.49-1.19)	0.82 (0.52-1.30)	46/726 (6.5)	0.81 (0.53-1.24)	0.85 (0.54-1.31)
		P=0.237	P=0.403		P=0.331	P=0.455
35-39	37/729 (5.1)	0.72 (0.46-1.14)	0.77 (0.48-1.22)	45/729 (6.5)	0.81 (0.53-1.23)	0.82 (0.53-1.28)
		P=0.157	P=0.264		P=0.318	P=0.388
40+	92/952 (9.6)	1.43 (0.98-2.09)	1.53 (1.01-2.31)	62/952 (6.4)	0.79 (0.54-1.17)	0.80 (0.51-1.25)
		P=0.062	P=0.045		P=0.246	P=0.328
**Sex**						
Male	223/3535 (6.4)	1	1	222/3535 (6.7)	1	1
Female	47/361 (12.3)	2.06 (1.46-2.90)	2.30 (1.60-3.29)	39/361 (10.4)	1.62 (1.12-2.34)	1.68 (1.14-2.47)
		P<0.001	P<0.001		P=0.010	P=0.009
**Rank**						
NCO¹ / Other rank	225/3113 (7.1)	1	1	212/3113 (7.1)	1	1
Officer	45/783 (5.4)	0.74 (0.53-1.03)	0.55 (0.39-0.78)	49/783 (6.2)	0.87 (0.62-1.20)	0.89 (0.63-1.24)
				P=0.076	P=0.001		P=0.389	P=0.483
**Engagement type**								
Regular	179/3204 (5.7)	1	1	193/3204 (6.2)	1	1		
Reservist	91/692 (11.9)	2.23 (1.67-2.97)	1.99 (1.46-2.73)	68/692 (10.1)	1.69 (1.23-2.31)	1.75 (1.23-2.50)		
		P<0.001	P<0.001		P=0.001	P=0.002		
**Role on deployment**								
Non-combat	209/2789 (7.1)	1	1	190/2789 (6.9)	1	1		
Combat	56/998 (6.2)	0.86 (0.63-1.18)	0.85 (0.61-1.19)	63/998 (6.7)	0.97 (0.71-1.32)	0.82 (0.59-1.14)		
		P=0.854	P=0.336		P=0.972	P=0.243		
**Traumatic deployment experiences**								
No experiences	15/370 (3.7)	1	1	14/370 (4.5)	1	1		
1-3 experiences	150/2182 (6.7)	1.86 (1.07-3.24)	2.00 (1.14-3.49)	128/2182 (5.9)	1.33 (0.73-2.43)	1.34 (0.73-2.44)		
		P=0.028	P=0.015		P=0.345	P=0.346		
4+ experiences	105/1283 (8.2)	2.34 (1.32-4.12)	2.74 (1.54-4.85)	113/1283 (8.9)	2.08 (1.14-3.79)	2.10 (1.13-3.90)		
		P=0.003	P=0.001		P=0.017	P=0.018		
**Marital Status**								
In a relationship	194/2961 (6.6)	1	1	198/2961 (3.9)	1	1		
Single	41/653 (6.3)	0.95 (0.66-1.38)	0.85 (0.58-1.25)	45/653 (7.1)	1.29 (0.72-1.47)	0.85 (0.58-1.24)		
		P=0.789	P=0.413		P=0.873	P=0.404		
Ex-relationship	33/267 (11.4)	1.84 (1.23-2.75)	1.57 (1.05-2.33)	18/267 (6.7)	0.97 (0.58-1.63)	0.98 (0.58-1.66)		
		P=0.003	P=0.027		P=0.914	P=0.944		

*Adjusted for age, sex, rank, engagement type and traumatic deployment experiences.

^1^NCO refers to non-commissioned officer.

**Test for trend P = 0.227.

***Test for trend P = 0.213.

Number of missing values ranges from 1–109.

### Association of events on OpEDAR and mental health problems

Having any event on OpEDAR was strongly associated with reporting ‘Fair to Poor’ general health (Table 3). Personnel medically evacuated for an illness or injury had over three times the odds of having ‘Fair to Poor’ general health (Table 4). Having any event on OpEDAR was strongly associated with increased risk of probable PTSD (Table 3). Personnel medically evacuated by air for an injury or illness event on OpEDAR had over four times the odds of having probable PTSD (Table 4).

**Table 3:** **The association between having any event on OpEDAR whilst deployed to Iraq and Afghanistan and subsequent mental health problems in Army personnel**

	**Alcohol Misuse**			**Fair to Poor GeneralHealth**			**Probable PTSD**			**Common Mental Disorders**		
	**Prevalence**	**Unadjusted OR**	Adjusted OR*	**Prevalence**	**Unadjusted OR**	Adjusted OR*	**Prevalence**	**Unadjusted OR**	Adjusted OR*	**Prevalence**	**Unadjusted OR**	Adjusted OR*
	**n (%)**	**(95% CI)**	**(95% CI)**	**n (%)**	**(95 % CI)**	**(95% CI)**	**n (%)**	**(95 % CI)**	**(95 % CI)**	**n (%)**	**(95 % CI)**	**(95 % CI)**
**No event on OpEDAR**	518/3312	1	1	367/3346	1	1	139/3330	1	1	633/3324	1	1
	(17.5)			(10.4)			(4.3)			(19.2)		
**Any event on OpEDAR**	87/521	1.10 (0.85-1.43)	1.15 (0.88-1.50)	94/528	1.70 (1.31-2.20)	1.73 (1.32-2.27)	41/526	1.88 (1.29-2.74)	1.57 (1.05-2.35)	154/520	1.68 (1.35-2.09)	1.54 (1.23-1.92)
	(18.9)	P=0.469	P=0.323	(16.5)	P<0.001	P<0.001	(7.9)	P=0.001	P=0.027	(28.6)	P<0.001	P<0.001

*Adjusted for age, sex, rank, engagement status, traumatic deployment experiences and marital status.

Number of missing values ranges from 22–63.

**Table 4:** **The association between having an illness or injury event on OpEDAR whilst deployed to Iraq or Afghanistan and subsequent mental health problems in UK Army personnel, where injuries and illnesses are categorised by disposal type**

		**Alcohol Misuse**			**Fair to Poor GeneralHealth**			**Probable PTSD**			**Common Mental Disorders**		
		**Prevalence**	**Unadjusted OR**	Adjusted OR*	**Prevalence**	**Unadjusted OR**	Adjusted OR*	**Prevalence**	**Unadjusted OR**	Adjusted OR*	**Prevalence**	**Unadjusted OR**	Adjusted OR*
		**n (%)**	**(95% CI)**	**(95% CI)**	**n (%)**	**(95% CI)**	**(95% CI)**	**n (%)**	**(95% CI)**	**(95% CI)**	**n (%)**	**(95% CI)**	**(95% CI)**
**No event on OpEDAR**		518/3312	1	1	367/3346	1	1	139/3330	1	1	633/3324	1	1
		(17.5)			(10.4)			(4.3)			(19.2)		
**Illness**	**Returned to unit**	9/73	0.74 (0.36-1.53)	0.78 (0.35-1.72)	15/74	1.94 (1.08-3.49)	1.79 (0.99-3.23)	7/75	2.00 (0.89-4.45)	1.58 (0.67-3.73)	22/74	1.74 (1.03-2.92)	1.57 (0.94-2.65)
		(13.6)	P=0.422	P=0.542	(18.5)	P=0.027	P=0.052	(8.3)	P=0.091	P=0.292	(29.3)	P=0.037	P=0.087
	**Admitted**	24/149	1.06 (0.66-1.70)	1.11 (0.68-1.81)	27/151	1.53 (0.98-2.37)	1.51 (0.96-2.37)	6/149	0.78 (0.34-1.82)	0.59 (0.25-1.41)	47/148	1.75 (1.21-2.54)	1.49 (1.01-2.20)
		(18.3)	P=0.819	P=0.672	(15.1)	P=0.059	P=0.077	(3.4)	P=0.573	P=0.239	(29.4)	P=0.003	P=0.045
	**Medically evacuated**	4/37	0.61 (0.21-1.81)	0.75 (0.23-2.51)	11/38	3.97 (1.89-8.32)	3.82 (1.78-8.23)	6/38	4.92 (1.88-1.82)	4.39 (1.60-12.07)	15/38	3.00 (1.50-6.00)	2.79 (1.41-5.51)
		(11.5)	P=0.375	P=0.644	(31.6)	P<0.001	P=0.001	(18.3)	P=0.001	P=0.004	(41.6)	P=0.002	P=0.003
**Injury**	**Returned to unit**	27/155	1.13 (0.73-1.75)	1.22 (0.78-1.91)	15/156	0.86 (0.49-1.51)	0.89 (0.51-1.57)	9/155	1.23 (0.60-2.52)	1.18 (0.57-2.47)	37/153	1.24 (0.83-1.85)	1.20 (0.79-1.80)
		(19.3)	P=0.594	P=0.390	(9.1)	P=0.597	P=0.695	(5.3)	P=0.571	P=0.654	(22.8)	P=0.286	P=0.392
	**Admitted**	11/47	1.80 (0.89-3.63)	1.78 (0.89-3.58)	6/47	1.13 (0.46-2.80)	1.27 (0.50-3.24)	4/47	2.34 (0.80-6.79)	1.79 (0.60-5.34)	13/45	1.63 (0.83-3.19)	1.52 (0.76-3.02)
		(27.6)	P=0.101	P=0.104	(11.6)	P=0.789	P=0.621	(9.6)	P=0.119	P=0.296	(27.9)	P=0.160	P=0.237
	**Medically evacuated**	9/49	1.45 (0.69-3.06)	1.23 (0.61-2.46)	16/50	3.56 (1.90-6.68)	3.88 (2.01-7.48)	8/50	5.00 (2.24-11.2)	4.27 (1.80-10.12)	14/50	1.65 (0.86-3.16)	1.51 (0.76-2.97)
		(23.6)	P=0.326	P=0.567	(29.3)	P<0.001	P<0.001	(18.5)	P<0.001	P=0.001	(28.2)	P=0.134	P=0.235

*Adjusted for age, sex, rank, engagement status, traumatic deployment experiences and marital status.

Number of missing values ranges from 34–74.

Having any event on OpEDAR was strongly associated with increased risk of symptoms of common mental disorders (Table 3). Personnel with an illness event on OpEDAR requiring admission or air-evacuation had over 1.5 and almost three times the odds, respectively, of having common mental disorders (Table 4). There was no association between common mental disorders and having an injury event on OpEDAR for any disposal type.

The only mental health problem not associated with having any event on OpEDAR was alcohol misuse (Tables 3 and 4).

### Hostile injuries

Individuals whose injury event on OpEDAR was hostile, compared to individuals with a non-hostile injury event, had 5 times the odds of having probable PTSD in unadjusted analysis [OR 5.00, 95% CI 1.80-13.88]; this remained after adjusting for age, sex, rank, engagement status and role on deployment [OR 3.88 95% CI 1.15-13.06]. Hostile injury events were not associated with any other mental health outcomes.

### Sensitivity analysis

Two sensitivity analyses were undertaken; the first where illness took precedence over injury for individuals with multiple OpEDAR events on their last deployment; and the second adjusting for OpEDAR events occurring before the last deployment. There were no notable differences in the associations between having an event on OpEDAR and subsequent mental health problems in either of these sensitivity analyses from the results presented here [data not shown; see Additional file 1]. When the sensitivity analysis excluding psychiatric cases (n = 7) was run, there were no notable differences in the results [data not shown; see Additional file 1]. The final sensitivity analysis included participants with prior mental health information from phase 1 (n = 2472); there were no major differences in the results [data not shown; see Additional file 1].

## Discussion

The main findings of this study are that as hypothesised, sustaining an injury on deployment is associated with over four times the odds of developing PTSD. Contrary to our hypothesis, having an illness on deployment that results in attendance at a field hospital is associated with post deployment mental health problems. The strength of this association is similar to, if not more than, the association with having a physical injury. Being returned to unit following attendance at a field hospital was not associated with any adverse effects on post deployment mental health. Attending a field hospital on deployment for either an illness or an injury was not associated with reported alcohol misuse post deployment.

It is widely accepted that serious injury increases the risk of probable PTSD [6,8,19] particularly injuries resulting from hostile action [20] and this has been corroborated in this current study. On the other hand, the finding that being medically evacuated for an illness was strongly associated with having probable PTSD and common mental disorders is a novel finding within the military literature. Evidence from civilian populations indicates that patients with chronic illnesses report symptoms of PTSD as do those requiring treatment in intensive care [21,22]. The perceived, and often actual, threat to life during episodes of these illnesses may trigger PTSD [21]. Illness has also been identified as a risk-factor for depression and anxiety in civilian populations, thought to be due to an increased pain and, or in addition, to it disabling the individual [9].

However, the illness events requiring medical air-evacuation are often not life-threatening or chronic; patients requiring prolonged treatment are routinely evacuated as field hospital space is limited and a substantially wider range of treatment options are available in the UK. It may be, instead, that the social environment faced by those returning home due to an illness puts them at greater risk of mental health problems in two ways. First, whereas injured personnel are recognised as being vulnerable to mental health problems, to the extent they have mandatory mental health monitoring, personnel with an illness are less visible and may feel more isolated; low levels of social support is a known risk factor for PTSD and other mental health conditions [23].

Second, personnel with illnesses may be subject to a greater degree of stigma than people with injuries. Society tends to treat personnel seriously injured on deployment as war heroes, with an ‘honourable’ reason for leaving, while those leaving the deployment due to illness may not receive the same degree of reverence and may be, or at least feel, stigmatised.

The other main finding is that personnel returned directly to their unit after attending an emergency department are not at increased risk of mental health problems post deployment. This fits with the UK military’s policy on treating psychiatric injuries known as ‘forward psychiatry’; that is, to treat them within the proximity of where the event is presented, to deliver care immediately and with the expectancy of occupational recovery [24]. By avoiding evacuation too readily and keeping personnel with their unit, evidence suggests that psychiatric problems are less likely to develop [24]. Military patients with general medical problems may also realise benefits to their mental health in the long term if, where their medical state allows, they are returned quickly to the support of colleagues and allowed to remain operationally effective. However, it should be considered that the severity of the injury or illness for those returned to unit is likely to be low, and it may be this factor making them less vulnerable to post deployment mental health problems.

This study also found that attending an emergency department for any reason, including a hostile injury, was not associated with an increased risk of alcohol misuse among UK Army personnel. Higher rates of alcohol misuse have been observed among US personnel exposed to ‘threatening situations’, one of which was ‘being injured or wounded’ [25] and mild traumatic brain injury (mTBI) has been associated with increased risk of alcohol misuse [26]. It may be that specific injuries, such as mTBI, increase the risk of alcohol misuse, but when combining all injuries no effect is found. The lack of association with illness may be explained by the illness itself not being conducive with drinking alcohol. Despite the questionnaire being self-reported and anonymous, alcohol use may be under-reported as excessive use is socially undesirable.

One of the main strengths of this study has been the ability to distinguish between illness and injury, allowing the impact of becoming ill during a military operation on post deployment mental health to be studied for the first time. The study also benefits from using routinely collected data on injuries and illnesses, rather than self-reported data, which many studies looking at injury and mental health rely on [6,27]. This reduces the potential of recall bias; specifically that those with and without mental health problems report injuries or illnesses experienced on deployment differently.

The current study has certain limitations. Injuries or illnesses occurring on deployment treated in primary care settings are unlikely to be captured by OpEDAR; further, accessibility to the field hospitals is likely to impact on field hospital attendance. This may explain the lack of association seen between injuries and role on deployment, since intuitively those in combat roles would be expected to have more injuries. Another limitation of the data is that severity was only assessed through a proxy (disposal type); some studies have found the risk of PTSD increases with the severity of the combat injury [20]. Additionally, the study is limited to Army personnel meaning the results are not generalisable to the entire UK military; this was justified as the Army are the largest group to deploy and non-Army personnel had a very small number of OpEDAR events. Identifying the direction of any bias introduced from 10% of the study sample being excluded due to non-consent of use of medical records is not possible. As the proportion excluded is small, it is unlikely to have a significant effect on the results reported here. Furthermore, although a sensitivity analysis was run where illness took precedence over injury, it is acknowledged that the associations found here may be affected by the choice of the most severe event. The authors also acknowledge that the illnesses experienced whilst on deployment may have been psychosomatic manifestations of the stress response. If a physician believes an illness to be psychosomatic, it may have been recorded as a psychiatric illness on OpEDAR, though it is appreciated that distinguishing physical illness from somatic symptoms caused by distress is challenging. However we believe this is unlikely to account for all the association between physical illness on deployment and post-deployment mental health problems observed here. Finally, it is acknowledged that the conclusions regarding the mental health consequences of air-evacuations for medical reasons rely on small numbers and must thus be treated with caution.

## Conclusions

The two main conclusions of this study are that first, personnel sustaining illnesses on deployment are just as, if not more, likely to report post deployment mental health problems as personnel who have sustained an injury. Second, personnel who were returned to unit did not have any increased reporting of post deployment mental health problems. These results suggest that monitoring of mental health problems should include those with illnesses, as well as those with injuries sustained on deployment.

## Competing interests

(1) NG is member of the Royal Naval Services and NJ is member of British Army. Although they are paid by the UK Ministry of Defence (MoD), they were not directed in any way by the MoD in relation to this publication. (2) S Wessely is Honorary Civilian Consultant Advisor in Psychiatry to the British Army and a Trustee of Combat Stress. (3) HJF and NTF are funded by the Ministry of Defence but they were not directed in any way by the MoD in relation to this publication. (4) KH and S White are civilian members of the MoD.

## Authors’ contributions

All authors read and approved the final manuscript. I, HJF, developed the analytical strategy for this paper, processed and analysed the data and wrote the paper. I agree with: the contents of the manuscript; and to being listed as an author. I have had access to all the data in the study and accept responsibility for its validity. I am a guarantor of this study. I, NJ, provided military assistance and advice on the design of the KCMHR cohort study, on the data processing for this analysis, and have commented on the paper. I agree with: the contents of the manuscript; and to being listed as an author. I, CW, was involved in discussing the data processing and analysis of the data, as well as the writing of the paper. I agree with: the contents of the manuscript; and to being listed as an author. I, NG, provided military assistance and advice in the design and undertaking of the KCMHR cohort study, and have commented on the paper. I agree with: the contents of the manuscript; and to being listed as an author. I, KH, was involved in discussions of the analytical approach to this study, supplied the OpEDAR data and made comments on the analysis and the writing of the paper. I agree with: the contents of the manuscript; and to being listed as an author. I, SW, was involved in discussions of the analytical approach to this study, supplied the OpEDAR data and made comments on the analysis and the writing of the paper. I agree with: the contents of the manuscript; and to being listed as an author. I, SW, am the chief investigator for the KCMHR cohort study, I was responsible for securing funding for this study and I led the design and planning of the study. I have commented on the paper. I agree with: the contents of the manuscript; and to being listed as an author. I, NF, am one of the principal investigators for the KCMHR cohort study, I was involved in the design and planning of the study. I was involved in developing the analytical strategy for this paper, and I have commented extensively on the paper. I agree with: the contents of the manuscript; and to being listed as an author. I am the main guarantor of this study.

## Funding

The study was funded by the UK Ministry of Defence. The work was independent of the funders but a copy of the paper was sent to them at the point of submission. The Defence Analytical Services and Advice provided the sampling frames of the Armed Forces and the Operational Emergency Department Attendance Register data. The funders did not participate in data collection, data processing, data analysis, or interpretation of findings.

## Pre-publication history

The pre-publication history for this paper can be accessed here:

http://www.biomedcentral.com/1471-244X/12/178/prepub

## Supplementary Material

Additional file 1 **Table A. Differences between consenters and non-consenters.** Table B1 Sensitivity analysis; the association between having an illness or injury event on OpEDAR whilst deployed to Iraq or Afghanistan and subsequent mental health problems in UK Army personnel, where illness takes precedent over injury. Table B2 Sensitivity analysis; the association between having an illness or injury event on OpEDAR whilst deployed to Iraq or Afghanistan and subsequent mental health problems in UK Army personnel, adjusting for having had a hospital attendance before their most recent deployment. Table B3 Sensitivity analysis; the association between having an illness or injury event on OpEDAR whilst deployed to Iraq or Afghanistan and subsequent mental health problems in UK Army personnel, removing OpEDAR events classified as “psychiatric illnesses”. Table B4 Sensitivity analysis; the association between having an illness or injury event on OpEDAR whilst deployed to Iraq or Afghanistan and subsequent mental health problems in UK Army personnel, adjusting for phase 1 mental health. (DOCX 36 kb) Click here for file
