# Supplementary material for: What are the effects of having an illness or injury whilst deployed on post deployment mental health? A population based record linkage study of UK Army personnel who have served in Iraq or Afghanistan
Source: BMC Psychiatry. 2012 Oct 24;12:178. doi: 10.1186/1471-244X-12-178 (PMC3507752; doi:10.1186/1471-244X-12-178)
Supplement: Additional file 1 — Table A. Differences between consenters and non-consenters. Table B1 Sensitivity analysis; the association between having an illness or injury event on OpEDAR whilst deployed to Iraq or Afghanistan and subsequent mental health problems in UK Army personnel, where illness takes precedent over injury. Table B2 Sensitivity analysis; the association between having an illness or injury event on OpEDAR whilst deployed to Iraq or Afghanistan and subsequent mental health problems in UK Army personnel, adjusting for having had a hospital attendance before their most recent deployment. Table B3 Sensitivity analysis; the association between having an illness or injury event on OpEDAR whilst deployed to Iraq or Afghanistan and subsequent mental health problems in UK Army personnel, removing OpEDAR events classified as “psychiatric illnesses”. Table B4 Sensitivity analysis; the association between having an illness or injury event on OpEDAR whilst deployed to Iraq or Afghanistan and subsequent mental health problems in UK Army personnel, adjusting for phase 1 mental health. (DOCX 36 kb) [file 1471-244X-12-178-S1.docx]

**Additional file**

Table A: Differences between consenters and non-consenters.

Table B1**:** Sensitivity analysis; the association between having an illness or injury event on OpEDAR whilst deployed to Iraq or Afghanistan and subsequent mental health problems in UK Army personnel, where illness takes precedent over injury.

Table B2**:** Sensitivity analysis; the association between having an illness or injury event on OpEDAR whilst deployed to Iraq or Afghanistan and subsequent mental health problems in UK Army personnel, adjusting for having had a hospital attendance before their most recent deployment.

Table B3**:** Sensitivity analysis; the association between having an illness or injury event on OpEDAR whilst deployed to Iraq or Afghanistan and subsequent mental health problems in UK Army personnel, removing OpEDAR events classified as “psychiatric illnesses”.

Table B4: Sensitivity analysis; the association between having an illness or injury event on OpEDAR whilst deployed to Iraq or Afghanistan and subsequent mental health problems in UK Army personnel, adjusting for phase 1 mental health.

**Table A:** Differences between consenters and non-consenters

|  | **Consenters** | **Non- consenters** | **P value*** |
| --- | --- | --- | --- |
| **Age group (years)** | | | |
| <25 | 657 (24.7) | 127 (45.8) | <0.001 |
| 25-29 | 832 (24.0) | 101 (29.6) |  |
| 30-34 | 726 (16.9) | 54 (14.0) |  |
| 35-39 | 729 (15.4) | 32 (7.6) |  |
| 40+ | 952 (19.0) | 15 (3.0) |  |
| **Sex** | | | |
| Male | 3535 (92.3) | 283 (86.0) | 0.034 |
| Female | 361 (7.7) | 46 (10.7) |  |
| **Rank** | | | |
| Non-Commissioned Officer / Other rank | 3113 (84.9) | 273 (87.8) | 0.106 |
| Commissioned Officer | 783 (15.1) | 56 (12.2) |  |
| **Marital status at time of questionnaire** | | | |
| Married/Cohabiting/Long term relationship | 2961 (73.8) | 213 (63.9) | <0.001 |
| Single | 653 (20.0) | 96 (32.4) |  |
| Separated / Divorced | 267 (6.1) | 13 (3.7) |  |
| **Serving status at time of questionnaire** | | | |
| Serving | 3158 (82.7) | 310 (95.1) | <0.001 |
| Discharged | 732 (17.3) | 14 (4.3) |  |
| **Traumatic deployment experiences** | | | |
| No experiences | 370 (9.0) | 21 (5.5) | <0.001 |
| 1-3 experiences | 2182 (55.3) | 158 (48.4) |  |
| 4 or more experiences | 1283 (35.6) | 137 (46.1) |  |
| **Role on deployment** | | | |
| Combat | 998 (29.1) | 99 (33.2) | 0.140 |
| Non-combat | 2789 (70.9) | 223 (66.8) |  |
| **Engagement status** | | | |
| Regular | (82.1) | 294 (87.0) | 0.054 |
| Reservist | (17.9) | 35 (13.0) |  |
| **Health outcomes** | | | |
| Alcohol Misuse | 605 (17.7) | 55 (19.4) | 0.464 |
| Fair to Poor general health | 461 (11.3) | 27 (8.4) | 0.125 |
| Probable PTSD | 180 (4.8) | 11 (3.6) | 0.376 |
| Common Mental Disorders | 787 (20.5) | 48 (15.0) | 0.026 |

*Using Pearson Chi-squared statistic

**Table B1.** The association between having an illness or injury event on OpEDAR whilst deployed to Iraq or Afghanistan and subsequent mental health problems in UK Army personnel, where illness takes precedent over injury.

|  | | **Alcohol Misuse** | | | **Fair/Poor General Health** | | | **Probable PTSD** | | | **Common Mental Disorders** | | |
| --- | --- | --- | --- | --- | --- | --- | --- | --- | --- | --- | --- | --- | --- |
|  | | Prevalence  n (%) | Unadjusted OR (95% CI) | Adjusted OR*  (95% CI) | Prevalence  n (%) | Unadjusted OR (95% CI) | Adjusted OR*  (95% CI) | Prevalence  n (%) | Unadjusted OR (95% CI) | Adjusted OR**  (95% CI) | Prevalence  n (%) | Unadjusted  OR (95% CI) | Adjusted OR*  (95% CI) |
| **No OPEDAR event** | | 518/3312  (17.5) | 1.00 | 1.00 | 367/3346 (10.4) | 1.00 | 1.00 | 139/3330 (4.3) | 1.00 | 1.00 | 633/3324  (19.2) | 1.00 | 1.00 |
| **Illness** | **Returned to unit** | 11/89  (14.1) | 0.77  (0.40-1.49) | 0.80 (0.40-1.62)  P=0.538 | 18/90  (17.4) | 1.81  (11.06-3.10) | 1.69 (0.98-2.92)  P=0.058 | 10/90  (9.6) | 2.34  (1.18-4.65) | 1.94 (0.94-3.99)  P=0.071 | 29/88  (33.2) | 2.09  (1.31-3.33) | 1.90 (1.19-3.04)  P=0.008 |
|  | **Admitted** | 25/156  (18.4) | 1.06 (0.67-1.69) | 1.03 (0.64-1.67)  P=0.893 | 27/158  (14.4) | 1.45  (0.93-2.24) | 1.44 (0.92-2.24)  P=0.111 | 7/156  (4.1) | 0.95  (0.43-2.10) | 0.73 (0.32-1.65)  P=0.452 | 49/155  (29.5) | 1.75  (1.22-2.52) | 1.57 (1.08-2.28)  P=0.019 |
|  | **Medically Evacuated** | 4/37  (11.2) | 0.61 (0.21-1.81) | 0.68 (0.21-2.22)  P=0.529 | 11/38  (31.6) | 3.67  (1.89-8.32) | 3.77 (1.75-8.13)  P=0.001 | 6/38  (18.3) | 4.92  (1.88-12.9) | 4.44 (1.62-12.16)  P=0.004 | 15/38  (41.6) | 3.00  (1.50-6.00) | 2.82 (1.43-5.55)  P=0.003 |
|  | | | | | | | | | | | | | |
| **Injury** | **Returned to unit** | 25/139  (19.7) | 1.15  (0.73-1.82) | 1.25 (0.78-2.00)  P=0.345 | 12/140  (8.6) | 0.81  (0.43-1.51) | 0.85 (0.46-1.58)  P=0.609 | 6/140  (4.2) | 0.97  (0.41-2.29) | 0.95 (0.40-2.27)  P=0.905 | 15/38  (20.0) | 1.04  (0.68-1.61) | 1.03 (0.66-1.59)  P=0.911 |
|  | **Admitted** | 10/40  (28.9) | 1.91  (0.91-4.03) | 1.83 (0.85-3.96)  P=0.121 | 6/40  (13.7) | 1.37  (0.55-3.42) | 1.45 (0.56-3.75)  P=0.449 | 3/40  (8.0) | 1.91  (0.56-6.53) | 1.47 (0.42-5.15)  P=0.546 | 30/139  (27.5) | 1.59  (0.76-3.30) | 1.44 (0.71-2.94)  P=0.311 |
|  | **Medically Evacuated** | 9/49  (23.6) | 1.45  (0.69-3.06) | 1.22 (0.58-2.55)  P=0.598 | 16/50  (29.3) | 3.56  (1.90-6.68) | 3.83 (2.00-7.32)  P<0.001 | 8/50  (18.5) | 5.00  (2.24-11.16) | 4.26 (1.81-10.01)  P=0.001 | 11/38  (28.2) | 1.65  (0.86-3.16) | 1.51 (0.78-2.95)  P=0.222 |

*Adjusted for age, sex, rank, engagement status and traumatic deployment experiences

**Table B2:** The association between having an illness or injury event on OpEDAR whilst deployed to Iraq or Afghanistan and subsequent mental health problems in UK Army personnel, adjusting for having had a hospital attendance before their most recent deployment.

|  | | **Alcohol Misuse** | | | **Fair/Poor General Health** | | | **Probable PTSD** | | | **Common Mental Disorders** | | |
| --- | --- | --- | --- | --- | --- | --- | --- | --- | --- | --- | --- | --- | --- |
|  | | Prevalence  n (%) | Unadjusted OR (95% CI) | Adjusted OR*  (95% CI) | Prevalence  n (%) | Unadjusted OR (95% CI) | Adjusted OR*  (95% CI) | Prevalence  n (%) | Unadjusted OR (95% CI) | Adjusted OR**  (95% CI) | Prevalence  n (%) | Unadjusted  OR (95% CI) | Adjusted OR*  (95% CI) |
| **No OPEDAR event** | | 518/3312 (17.5) | 1 | 1 | 367/3346 (10.4) | 1 | 1 | 139/3330 (4.3) | 1 | 1 | 633/3324 (19.2) | 1 |  |
| **Illness** | **Returned to unit** | 9/73 (13.6) | 0.74 (0.36-1.53)  P=0.422 | 0.82 (0.37-1.82)  P=0.631 | 15/74  (18.5) | 1.94 (1.08-3.49)  P=0.027 | 1.81 (1.00-3.29)  P=0.051 | 7/75 (8.3) | 2.00 (0.89-4.45)  P=0.091 | 1.61 (0.70-3.70)  P=0.260 | 22/74 (29.3) | 1.74 (1.03-2.92)  P=0.037 | 1.59 (0.94-2.67)  P=0.082 |
|  | **Admitted** | 24/149 (18.3) | 1.06 (0.66-1.70)  P=0.819 | - 1. (0.63-1.69)   P=0.898 | 27/151 (15.1) | 1.53 (0.98-2.37)  P=0.059 | 1.50 (0.96-2.34)  P=0.076 | 6/149 (3.4) | 0.78 (0.34-1.82)  PP=0.573 | 0.60 (0.25-1.43)  P=0.251 | 47/148 (29.4) | 1.75 (1.21-2.54)  P=0.003 | 1.56 (1.07-2.29)  P=0.022 |
|  | **Medically Evacuated** | 4/37 (11.5) | 0.61 (0.21-1.81)  P=0.375 | 0.69 (0.21-2.24)  P=0.536 | 11/38  (31.6) | 3.97 (1.89-8.32)  P<0.001 | 3.80 (1.76-8.23)  P=0.001 | 6/38 (18.3) | 4.92 (1.88-1.82)  P=0.001 | 4.42 (1.62-12.03)  P=0.004 | 15/38 (41.6) | 3.00 (1.50-6.00)  P=0.002 | 2.83 (1.43-5.59)  P=0.003 |
|  | | | | | | | | | | | | | |
| **Injury** | **Returned to unit** | 27/155 (19.3) | 1.13 (0.73-1.75)  P=0.594 | 1.18 (0.75-1.85)  P=0.480 | 15/156  (9.1) | 0.86 (0.49-1.51)  P=0.597 | 0.88 (0.50-1.55)  P=0.653 | 9/155 (5.3) | 1.21 (0.60-2.52)  P=0.571 | 1.20 (0.58-2.51)  P=0.623 | 37/153 (22.8) | 1.24 (0.83-1.85)  P=0.286 | 1.21 (0.76-2.92)  P=0.354 |
|  | **Admitted** | 11/47 (27.6) | 1.80 (0.89-3.63)  P=0.101 | 1.70 (0.82-3.50)  P=0.151 | 6/47  (11.6) | 1.13 (0.46-2.80)  P=0.789 | 1.24 (0.48-3.17)  P=0.653 | 4/47 (9.6) | 2.34 (0.80-6.79)  P=0.119 | 1.77 (0.60-5.20)  P=0.300 | 13/45 (27.9) | 1.63 (0.83-3.19)  P=0.160 | 1.49 (0.76-2.92)  P=0.248 |
|  | **Medically Evacuated** | 9/49 (23.6) | 1.45 (0.69-3.06)  P=0.326 | 1.23 (0.59-2.58)  P=0.581 | 16/50  (29.3) | 3.56 (1.90-6.68)  P<0.001 | 3.86 (2.03-7.36)  P<0.001 | 8/50 (18.5) | 5.00 (2.24-11.2)  P<0.001 | 4.19 (1.77-9.92)  P=0.001 | 14/50 (28.2) | 1.65 (0.86-3.16)  P=0.134 | 1.52 (0.78-2.96)  P=0.215 |

*Adjusted for age, sex, rank, engagement status and traumatic deployment experiences having had a hospital attendance before most recent deployment

Table B3: Sensitivity analysis; the association between having an illness or injury event on OpEDAR whilst deployed to Iraq or Afghanistan and subsequent mental health problems in UK Army personnel, removing OpEDAR events classified as “psychiatric illnesses”.

|  | | **Alcohol Misuse** | | | **Fair to Poor General Health** | | | **Probable PTSD** | | | **Common Mental Disorders** | | |
| --- | --- | --- | --- | --- | --- | --- | --- | --- | --- | --- | --- | --- | --- |
|  | | Prevalence  n (%) | Unadjusted OR (95% CI) | Adjusted OR*  (95% CI) | Prevalence  n (%) | Unadjusted OR (95% CI) | Adjusted OR*  (95% CI) | Prevalence  n (%) | Unadjusted OR (95% CI) | Adjusted OR*  (95% CI) | Prevalence  n (%) | Unadjusted  OR (95% CI) | Adjusted OR*  (95% CI) |
| **No event on OpEDAR** | | 518/3312 (17.5) | 1 | 1 | 367/3346 (10.4) | 1 | 1 | 139/3330 (4.3) | 1 | 1 | 633/3324 (19.2) | 1 | 1 |
| **Illness** | **Returned to unit** | 7/72 (13.9) | 0.76 (0.37-1.56)  P=0.451 | 0.82 (0.37-1.80)  P=0.612 | 14/73  (17.2) | 1.78 (0.98-3.26)  P=0.060 | 1.64 (0.90-3.00)  P=0.106 | 7/74 (8.4) | 2.03 (0.91-4.53)  P=0.084 | 1.64 (0.69-3.86)  P=0.260 | 21/73  (28.2) | 1.65 (0.97-2.79)  P=0.064 | 1.50 (0.88-2.55)  P=0.135 |
|  | **Admitted** | 24/147 (18.6) | 1.08 (0.67-1.73)  P=0.757 | 1.14 (0.70-1.86)  P=0.596 | 27/149 (15.4) | 1.56 (1.00-2.42)  P=0.049 | 1.53 (0.97-2.41)  P=0.069 | 6/147 (3.5) | 0.80 (0.34-1.85)  P=0.599 | 0.61 (0.25-1.45)  P=0.261 | 46/146 (29.3) | 1.74 (1.20-2.53)  P=0.004 | 1.48 (1.00-2.20)  P=0.051 |
|  | **Medically evacuated** | 4/33 (12.6) | 0.68 (0.23-2.02)  P=0.486 | 0.80 (0.24-2.71)  P=0.722 | 10/34  (32.3) | 4.10 (1.89-8.92)  P<0.001 | 4.07 (1.82-9.10)  P=0.001 | 6/34 (19.9) | 5.48 (2.07-14.49)  P=0.001 | 4.84 (1.74-13.46)  P=0.002 | 14/34 (43.6) | 3.24 (1.57-6.71)  P=0.002 | 3.09 (1.52-6.27)  P=0.002 |
|  | | | | | | | | | | | | | |
| **Injury** | **Returned to unit** | 27/155 (19.3) | 1.13 (0.73-1.75)  P=0.594 | 1.22 (0.78-1.91)  P=0.390 | 15/156  (9.1) | 0.86 (0.49-1.51)  P=0.597 | 0.89 (0.51-1.57)  P=0.695 | 9/155 (5.3) | 1.23 (0.60-2.52)  P=0.571 | 1.18 (0.57-2.47)  P=0.654 | 37/153 (22.8) | 1.24 (0.83-1.85)  P=0.286 | 1.20 (0.80-1.80)  P=0.392) |
|  | **Admitted** | 11/47 (27.6) | 1.80 (0.89-3.63)  P=0.101 | 1.78 (0.89-3.58)  P=0.104 | 6/47  (11.6) | 1.13 (0.46-2.80)  P=0.789 | 1.27 (0.50-3.24)  P=0.621 | 4/47 (9.6) | 2.34 (0.80-6.79)  P=0.119 | 1.79 (0.60-5.34)  P=0.296 | 13/45 (27.9) | 1.63 (0.83-3.19)  P=0.160 | 1.52 (0.76-3.02)  P=0.237 |
|  | **Medicallyevacuated** | 9/49 (23.6) | 1.45 (0.69-3.06)  P=0.326 | 1.23 (0.61-2.46)  P=0.567 | 16/50  (29.3) | 3.56 (1.90-6.68)  P<0.001 | 3.88 (2.01-7.48)  P<0.001 | 8/50 (18.5) | 5.00 (2.24-11.16)  P<0.001 | 4.27 (1.80-10.12)  0.001 | 14/50 (28.2) | 1.65 (0.86-3.16)  P=0.134 | 1.51 (0.77-2.98)  P=0.235 |

*Adjusted for age, sex, rank, engagement status, traumatic deployment experiences and marital status

Table B4: Sensitivity analysis; the association between having an illness or injury event on OpEDAR whilst deployed to Iraq or Afghanistan and subsequent mental health problems in UK Army personnel, adjusting for phase 1 mental health (N=2472)

|  | | **Alcohol Misuse** | | | **Fair to Poor General Health** | | | **Probable PTSD** | | | **Common Mental Disorders** | | | |
| --- | --- | --- | --- | --- | --- | --- | --- | --- | --- | --- | --- | --- | --- | --- |
|  | | Prevalence  n (%) | Unadjusted OR (95% CI) | Adjusted OR*  (95% CI) | Prevalence  n (%) | Unadjusted OR (95% CI) | Adjusted OR*  (95% CI) | Prevalence  n (%) | Unadjusted OR (95% CI) | Adjusted OR*  (95% CI) | Prevalence  n (%) | Unadjusted  OR (95% CI) | | Adjusted OR*  (95% CI) |
| **No event on OpEDAR** | | 287/2314  (13.1) | 1 | 1 | 298/2328  (12.8) | 1 | 1 | 102/2321  (4.7) | 1 | 1 | 439/2316  (19.2) | 1 | | 1 |
| **Illness** | **Returned to unit** | 3/26  (12.3) | 0.93 (0.28-3.14)  P=0.908 | 0.76 (0.20-2.88)  P=0.688 | 7/27  (26.4) | 2.43 (1.01-5.83)  P=0.047 | 2.14 (0.88-5.22)  P=0.095 | 1/27  (3.4) | 0.73 (0.10-5.41)  P=0.754 | 0.45 (0.05-3.77)  P=0.464 | 10/27  (38.2) | 2.61 (1.18-5.78)  P=0.018 | | 2.11 (1.00-4.45)  P=0.049 |
|  | **Admitted** | 4/24  (16.1) | 1.27 (0.43-3.79)  P=0.662 | 0.78 (0.20-3.02)  P=0.719 | 5/24  (21.4) | 1.85 (0.68-5.05)  P=0.230 | 2.18 (0.76-6.21)  P=0.146 | 1/24  (3.8) | 0.81 (0.11-6.07)  P=0.838 | 0.71 (0.07-6.88)  P=0.770 | 9/24  (40.6) | 2.89 (1.24-6.69)  P=0.014 | | 2.83 (1.12-7.10)  P=0.027 |
|  | **Medically evacuated** | 2/7  (28.5) | 2.64 (0.50-13.83)  P=0.251 | 3.39 (0.76-15.13)  P=0.109 | 0/7 | (no observations) | (no observations) | 2/7  (3.2) | 9.50 (1.81-49.7)  P=0.008 | 11.97 (1.84-77.81)  P=0.009 | 3/7  (44.3) | 3.36 (0.74-15.22)  P=0.115 | | 2.93 (0.63-13.52)  P=0.168 |
|  | | | | | | | | | | | | | | |
| **Injury** | **Returned to unit** | 6/43  (14.5) | 1.12 (0.46-2.70)  P=0.803 | - 1. (0.44-2.32)   P=0.984 | 1/43  (2.1) | 0.15 (0.02-1.08)  P=0.060 | 0.14 (0.02-1.08)  P=0.060 | 2/43  (5.4) | 1.17 (0.27-4.95)  P=0.835 | 1.06 (0.25-4.51)  P=0.933 | 10/43  (22.9) | | 1.25 (0.61-2.57)  P=0.542 | 1.16 (0.46-2.96)  P=0.749 |
|  | **Admitted** | 1/12  (7.2) | 0.51 (0.06-4.00)  P=0.524 | 0.71 (0.09-5.81)  P=0.752 | 1/12  (7.2) | 0.53 (0.07-4.10)  P=0.540 | 0.80 (0.11-5.91)  P=0.824 | 1/12  (7.2) | 1.59 (0.20-12.44)  P=0.660 | 4.36 (0.53-35.66)  P=0.169 | 2/12  (13.9) | | 0.68 (0.15-3.13)  P=0.622 | 1.13 (0.28-4.52)  P=0.867 |
|  | **Medicallyevacuated** | 2/10  (23.7) | 2.05 (0.43-9.76)  P=0.365 | 1.29 (0.30-5.49)  P=0.729 | 4/10  (38.7) | 4.28 (1.18-15.47)  P=0.027 | 4.01 (0.93-17.33)  P=0.063 | 2/10  (21.0) | 5.45 (1.13-26.38)  P=0.035 | 6.98 (1.38-35.35)  P=0.019 | 3/10  (29.2) | | 1.74 (0.44-6.82)  P=0.427 | 1.32 (0.26-6.53)  P=0.738 |

*Adjusted for age, sex, rank, engagement status, traumatic deployment experiences, marital status and phase 1 mental health
